# On the Constitution and Functions of the Cervix Uteri

**Published:** 1847-03

**Authors:** 


*On the Constitution and Functions of the Cervix Uteri.* By M. Negrier.—The following are the most important conclusions which M. N. draws from his investigations. The superior orifice of the cervix endeavours to contract powerfully the instant that the dilating body, whether the ovum or the fœtal head, has passed out of it; the superior orifice alone may oppose an obstacle to delivery, it alone may incarcerate the placenta, whereas the inferior orifice, when it has been fully and slowly dilated, returns to its ordinary size after a considerable time, and gradually. In females who are delivered for the first time, the orifices of the cervix contract before the walls of its cavity; and in women who have been delivered more than once, the lips of the external orifice close more slowly than the cavity of the cervix; and in all women the parietes of the cervix are thrown into folds from above downwards. These perpendicular folds result from the closure of the superior orifice, and are not entirely effaced even ten days after delivery. In cervical implantation of the placenta, M. N. seems to prefer plugging far before any other mode of treament. —*Ibid, from Arch. Gen. de Med.*

